# A Comprehensive Benchmark of Transcriptomic Biomarkers for Immune Checkpoint Blockades

**DOI:** 10.3390/cancers15164094

**Published:** 2023-08-14

**Authors:** Hongen Kang, Xiuli Zhu, Ying Cui, Zhuang Xiong, Wenting Zong, Yiming Bao, Peilin Jia

**Affiliations:** 1 CAS Key Laboratory of Genomic and Precision Medicine, Beijing Institute of Genomics, Chinese Academy of Sciences and China National Center for Bioinformation, Beijing 100101, China; 2 University of Chinese Academy of Sciences, Beijing 100049, China; 3 National Genomics Data Center, Beijing Institute of Genomics, Chinese Academy of Sciences and China National Center for Bioinformation, Beijing 100101, China

**Keywords:** immune checkpoint blockade (ICB), transcriptomic biomarkers, benchmark, ICB-portal, TIDE

## Abstract

**Simple Summary:**

Immune checkpoint blockades (ICBs) therapy has produced durable clinical responses in many cancer types, but only a fraction of patients can benefit from ICB treatment. Previous studies have reported multiple transcriptomic biomarkers to predict ICB responses and improve treatment precision in various cancer types. However, a timely and unbiased assessment of these biomarkers has yet to be conducted due to the lack of large-scale uniformly curated ICB-treated datasets. To address the needs, we developed ICB-Portal, a comprehensive resource about ICB including RNA-seq data of 29 datasets from public sources and standardized metadata of each study through a uniform pre-processing, 48 biomarker scores associated with ICB response, results of a systematic benchmark analysis evaluating the efficacy, and generalization ability for each biomarker in various scenarios such as different cancer types, anti-bodies, biopsy time, and combinatory treatments with other drugs by a standardized bioinformatics workflow and an online benchmark platform.

**Abstract:**

Immune checkpoint blockades (ICBs) have revolutionized cancer therapy by inducing durable clinical responses, but only a small percentage of patients can benefit from ICB treatments. Many studies have established various biomarkers to predict ICB responses. However, different biomarkers were found with diverse performances in practice, and a timely and unbiased assessment has yet to be conducted due to the complexity of ICB-related studies and trials. In this study, we manually curated 29 published datasets with matched transcriptome and clinical data from more than 1400 patients, and uniformly preprocessed these datasets for further analyses. In addition, we collected 39 sets of transcriptomic biomarkers, and based on the nature of the corresponding computational methods, we categorized them into the gene-set-like group (with the self-contained design and the competitive design, respectively) and the deconvolution-like group. Next, we investigated the correlations and patterns of these biomarkers and utilized a standardized workflow to systematically evaluate their performance in predicting ICB responses and survival statuses across different datasets, cancer types, antibodies, biopsy times, and combinatory treatments. In our benchmark, most biomarkers showed poor performance in terms of stability and robustness across different datasets. Two scores (TIDE and CYT) had a competitive performance for ICB response prediction, and two others (PASS-ON and EIGS_ssGSEA) showed the best association with clinical outcome. Finally, we developed ICB-Portal to host the datasets, biomarkers, and benchmark results and to implement the computational methods for researchers to test their custom biomarkers. Our work provided valuable resources and a one-stop solution to facilitate ICB-related research.

## 1. Introduction

Immune checkpoint blockades (ICBs) include monoclonal antibodies that target PD-1, PD-L1, and CTLA-4, among others. ICB has generated durable responses across many cancer types [1,2]. However, only a fraction of cancer patients has benefited from ICB. The clinical outcome to ICB treatment has been reported to be quite inconsistent in different cancer types, or even in different patients of the same cancer type. The incidence of immune-related adverse events (irAEs) and the considerable costs present a pressing challenge for ICB treatment selection [3,4]. Thus, it is critical to develop predictive biomarkers of ICB response to improve treatment precision. Previous studies have revealed that the genomic biomarkers are associated with ICB response in various cancer types [5,6,7,8,9,10,11], such as tumor mutation burden (TMB), microsatellite instability (MSI), somatic copy number alterations, and mutations in genes related to neoantigens, DNA repair, antigen presentation, and oncogenic pathways [12]. However, genomic factors alone are not sufficient to predict ICB responses [13]. In contrast, gene expression data can be used to infer the heterogeneous cell populations and molecular interactions of the tumor microenvironment (TME). Thus, gene expression data have been widely studied as candidate biomarkers for ICB response. 

Over the past years, many transcriptomic biomarkers along with corresponding computational methods have been reported to predict ICB response. By reviewing these methods, we generally categorized them as belonging to the gene-set-like group and the deconvolution-like group. The gene-set-like group often relies on a list of marker genes. These methods can be further categorized as ones that adapt the self-contained hypothesis and ones that adapt the competitive hypothesis. The self-contained methods use marker genes to calculate an overall score that can be further used to distinguish samples, e.g., responsive or non-responsive. Such methods do not rely on the non-marker genes in the transcriptome. The competitive methods calculate an overall score by using the ranks of marker genes as compared with non-marker genes in the transcriptome, such as single sample gene set enrichment analysis (ssGSEA). ssGSEA was first successfully implemented to infer the tumor purity and stromal and immune cell admixture across multiple cancer types [14]. By far, ssGSEA has been applied in many studies to investigate various biological processes including ICB response prediction. To incorporate genes with different functions during the immune therapy treatment, some methods combine scores from multiple sets to achieve the goal to distinguish samples. The deconvolution-like methods often rely on the deconvolutional analyses of the whole transcriptome, although some of them also require a pre-defined set of marker genes.

**Figure 1:**
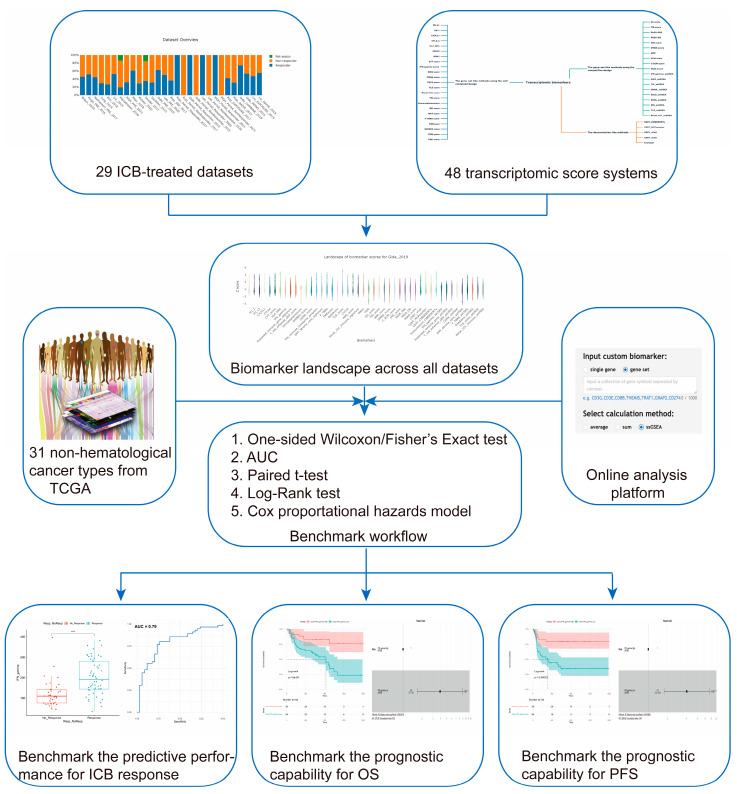
An overview of the study design. First, we manually curated the most comprehensive resource, containing 29 published datasets with matched transcriptome data and clinical information covering more than 1400 patients. In addition, we collected a total of 48 scoring systems from 39 sets of transcriptome biomarkers (top panels). Second, we built the biomarker landscape by computing the biomarker scores across 29 datasets (middle panels). Third, we utilized a standardized bioinformatics workflow to systematically evaluate these transcriptomic biomarkers for ICB response, overall survival (OS), and progression-free survival (PFS) across different datasets, cancer types, anti-bodies, biopsy times, and combinatory treatments with other drugs (bottom panels). Moreover, we validated the predictive power of biomarkers for OS by applying the biomarkers in 31 TCGA non-hematological cancer types. Finally, we provided an online analysis platform to benchmark the predictive performance of user-provided custom biomarkers on ICB response and the prognostic ability of OS and PFS based on the ICB-treated dataset contained in our database. ** *p* < 0.01, *** *p* < 0.001, **** *p* < 0.0001.

In this work, we manually curated 29 published datasets where the samples were treated with various ICB therapies (hereafter named ICB-treated samples). All these datasets had matched transcriptome data and clinical information for the benchmark task to evaluate the method performance in predicting ICB response. Furthermore, these datasets covered different cancer types, antibodies, biopsy times, and combinatory treatments with other drugs. Thus, they presented a comprehensive resource for future studies. We collected a total of 39 sets of transcriptomic biomarkers and methods, which generated 48 scoring systems, and systematically assessed their capability in predicting ICB response, overall survival (OS), and progression-free survival (PFS) across different datasets including both the ICB-treated datasets and the Cancer Genomics Atlas (TCGA). Finally, we organized these results into a database named ICB-Portal and provided an online analysis platform to allow users to benchmark their own biomarkers of interests in predicting ICB response (Figure 1). Altogether, these results will strengthen our understanding of fundamental molecular mechanisms of immunotherapy resistance and facilitate the discovery of novel therapeutic targets and potential predictors. All datasets, biomarkers, analysis results, and related resources can be found in ICB-Portal via https://ngdc.cncb.ac.cn/icb (accessed on 1 July 2023).

## 2. Materials and Methods

### 2.1. ICB Transcriptomic Biomarker and Method Collection

We conducted a systematic literature search and curation of transcriptomic biomarkers for ICB response. As a result, we collected a total of 39 sets transcriptomic biomarkers for ICB response prediction that can be generally divided into three categories according to the calculation methods: (1) the gene-set-like methods using the self-contained hypothesis which rely on a list of marker genes (*n* = 23); (2) the gene-set-like methods using the competitive hypothesis such as the ssGSEA-based methods (*n* = 10) or a permutation-based method (*n* = 1); and (3) the deconvolution-like methods (*n* = 5). In addition, for nine biomarkers originally used by the first group, i.e., the gene-set-like methods using the self-contained hypothesis, we further calculated the ssGSEA scores using these gene sets and additionally constructed 9 new scoring measurements. Thus, we have a total of 48 scoring systems from 39 sets of transcriptome biomarkers for the following benchmark analysis (Table 1). Below we review each of the methodological details of these methods, respectively.

### 2.2. The Gene-Set-Like Group Methods with Self-Contained Design

The self-contained methods use the expression of a list of marker genes (ranging from 1 to 162, except TIDE, see below). These marker genes are often determined by using a priori knowledge or previous studies. There are 23 methods belonging to this group.

Eight methods use a single gene as the marker based on different biological assumptions. *PD-L1* expression is the first predictive transcriptomic biomarker for the response of anti-PD-1 immunotherapy [15], although the expression level varies dramatically in different cancer types. *PD-L1* shows a high co-expression with *PD-1* and *PD-L2* and therefore, the three genes (*PD-L1*, *PD-1*, and *PD-L2*) are often used as marker genes for the anti-PD-1 immunotherapy [17,18]. Similarly, other single marker genes used for anti-PD-L1 response include *CTLA-4*, *CXCL1* [15], and *CXCL9* (a marker of tumor-associated macrophages (TAM) subset) [20]. In addition, the major histocompatibility complex class-I and -II (MHC-I and MHC-II) have been linked to tumor antigen presentation. Thus, the expression of *HLA-DRA*, which is the prototype MHC-II molecule, has been used as a biomarker for predicting the anti-PD-1 response [19]. Lastly, patients receiving ICB along with antihistamines have better survival outcome, likely due to the association between histamine receptor H1 (*HRH1*) with T-cell dysfunction, and thus, the gene *HRH1* has also been used as a marker to predict ICB response [21].

Seven methods used the average expression of multiple marker genes. The immune cytolytic activity score (CYT score) is used to measure cytotoxic T cells [22] and is computed as the mean expression of two critical effector molecules that mediate cytolysis, which are granzyme A (*GZMA*) and perforin (*PRF1*). The IFN-gamma score is calculated using the expression of six genes related to IFN-γ signaling and T-cell activities [23]. IFN-γ is a critical cytokine secreted by natural killer (NK) and T cells and can be used to predict the response to PD-1 blockade [23]. The expanded immune gene signature (EIGS) score [23] is calculated based on 18 related genes and can be used to predict the ICB response. The CRMA score is calculated as the geometric mean of the expression levels for 8 anti-CTLA-4 resistance associated MAGE-A genes (CRMA) [24]. The EMT/Stroma core signature (ESCS) score is calculated using the mean expression of 8 marker genes that correlate strongly with immune resistance to PD-1 blockade in urothelial cancer [25]. The tertiary lymphoid structure (TLS) signature score is calculated using the mean expression of 9 genes and has been used to predict the clinical outcomes in melanoma patients treated with ICB [46]. Finally, the renal 101 immune signature (Renal-101) score is calculated as the mean expression of 26 genes involved in innate immune responses, cell trafficking, and inflammation [27].

Four methods used a weighted sum of multiple marker genes. The T cell-inflamed gene expression profiles (TIG) score is calculated as the weighted sum of the expression of 18 signature genes [23]. The Immunophenoscore is calculated as the sum of the weighted average expression of the marker genes from four categories: MHC molecules, immunomodulators, effector cells, and suppressor cells [29]. The immune-related risk score (IRG) is calculated as the weighted sum of the expression of 11 immune-related genes and is predictive of survival and ICB response for patients with cervical cancer [30]. The melanocytic plasticity signature (MPS) score is calculated using the weighted sum of the expression of 45 genes. MPS score reflects the extent of differentiation or multipotency of melanocytic lineage and is predictive of ICB efficacy [31]. 

The remaining methods implement different forms of computation. The pan-fibroblast TGF-beta response signature (F-TBRS) and the TMEscore are both calculated using the principal component analysis (PCA) of the signature genes [32,33]. The immuno-predictive score (IMPRES) is calculated based on the transcriptomics relationship of 15 pairs of relationships among 15 immune checkpoint genes [34] and is initially developed as a predictor of ICB response in melanoma. Thus, IMPRES has a range between 0 and 15. Tumor immune dysfunction and exclusion (TIDE) is a computational framework to identify the gene signature related to immune escape and can serve as a biomarker of ICB response [35]. 

### 2.3. The Gene-Set-like Group METHODS with Competitive Design

A total of 11 methods can be classified as the gene-set-like methods with competitive design, including 10 based on ssGSEA and one named the immune resistance program score (TIRP). Specifically, ssGSEA calculates a normalized enrichment score (NES) by comparing the ranks of the genes in the signature with other genes in the transcriptome. ssGSEA adopts the competitive hypothesis design and can be used as a measurement of the overexpression of a signature gene list. We collected a total of 10 ssGSEA-based methods from literature. Among them, some used the NES from one single gene set while others used different forms of combinations of multiple gene sets, e.g., average, sum, or weighted sum of multiple NES scores. 

Three methods used the NES from a single set of genes. The antigen-presenting machinery (APM) score is defined as the NES of a set of antigen-presentation-related genes, as such genes have been reported to be associated with ICB response in ccRCC patients [37]. The C-ECM score is defined as the NES of a set of 58 cancer-associated ECM genes (C-ECM), which is associated with the activity of TGF-beta and the presence of cancer associated fibroblast (CAFs) [41]. The MHC-I association immunoscore (MIAS) is calculated as the NES of 100 genes created by a network approach and has been used as a predictor of ICB response for melanoma patients [42].

Six methods used different forms of combinations of multiple gene sets. The immune infiltration score (IIS score) and the T cell infiltration score (TIS score) are defined as the sum of NES scores from multiple signature gene sets, each of which represents different processes involved in anti-PD1 immunotherapy [37]. Innate anti-PD-1 resistance, a transcriptional signature referred to as the IPRES, consists of genes involved in ECM, cell adhesion, regulation of mesenchymal transition, angiogenesis, and wound healing and has been used as a biomarker to improve anti-PD-1 response in multiple tumor types [13]. Immune microenvironment score (IMS) has been developed from six gastric cancer cohorts and 27 TME cell signatures related to the overall survival status. IMS is calculated as the weighted sum of ssGSEA NES scores [39]. The pathway-based super signature (PASS) scores are developed by analyzing the corresponding pre-treatment (PASS-PRE) and on-treatment (PASS-ON) cohorts [38]. They are calculated as the weighted sum of ssGSEA NES scores of several related pathways associated with ICB response. 

A recent study defined four distinct TME subtypes by unsupervised clustering based on ssGSEA NES scores of 29 knowledge-based functional gene expression features that comprehensively described major functional components and immune, stromal, and other cellular populations. Because this method has been shown to be correlated with ICB responses [40], we included it in our analyses and referred it as the molecular functional tumor portrait (MFP) score. 

Finally, the TIRP score is defined as the overall expression of genes involved in malignant cell programs that are associated with T-cell exclusion [36]. It was originally developed based on single-cell RNA-sequencing data and has been successfully used in melanoma patients.

Furthermore, we used nine biomarkers (IFN-gamma, EIGS, TIG, CRMA, ESCS, F-TBRS, IRG, TLS, and Renam-101 signature) from the first class of methods and calculated ssGSEA as new score systems.

### 2.4. The Deconvolution-like Methods

The third class of biomarkers is constructed based on the deconvolution type of methods applied to gene expression matrix (*n* = 5). By far, the representative deconvolution-like methods include CIBERSORT, CIBERSORTx, microenvironment cell populations (MCP)-counter, and xCell [47,48,49,50]. These methods implement different matrix decomposition algorithms to infer the proportions of different cell types from bulk gene expression data. Thus, many studies use the deconvolutional methods to infer the proportions of immune cell types to subsequently predict ICB responses.

Previous studies have revealed that the baseline levels of CD8+ T cells is an important determinant of clinical response to anti-PD-1 [43,51]. Thus, different deconvolution-like methods have been used to infer the proportion of CD8+ T cells from bulk expression, such as CD8T_CIBERSORTx [49], CD8T_MCPcounter, and CD8T_xCell. In addition, the immunoscore was constructed based on the weighted sum of the percentages of 8 immune subsets derived by using CIBERSORTx and could be used to predict response to anti-PD-1 in melanoma [44].

Lastly, EcoTyper is a machine learning framework for characterizing the cell states and multicellular communities from expression data. The first step of EcoTyper is applying CIBERSORTx to deconvolute cell-type specific expression profiles from bulk RNA-seq data. Second, non-negative matrix factorization (NMF) is used to identify cell states. Third, EcoTyper identified ten multicellular communities with distinct cell-state co-occurrence patterns and have been reported to be associated with OS and ICB response [45]. 

### 2.5. Benchmark Design

We used the TCGA multi-omics data and ICB-treated data to evaluate the 39 sets of biomarkers and 48 scoring measurements. Most TCGA samples did not receive ICB therapies or had no information about ICB treatment. These data were used to assess the transcriptomic correlations among the biomarkers as well as the relationships between biomarkers and other biological characteristics, such as the TMB burden and the stroma and immune scores. The TCGA clinical data were used to evaluate the general performance of these scoring systems with the patient survival status without stratifications by ICB-response. In addition, we collected the ICB-treated datasets, where the included samples received at least one type of ICB treatment. These data had well-recorded clinical outcome from ICB treatment as well as the pre-treatment transcriptome data and thus, are used to evaluate the performance of the scoring system in distinguishing ICB responders and nonresponders. 

### 2.6. TCGA Dataset Collection and Benchmark

For TCGA data, we used the 31 non-hematological cancer types including approximately 8000 samples. We used the R packages *TCGAbiolinks* to download the mutation data [52] and *Maftools* to calculate the TMB burden [53] for each sample. The TCGA RNA-seq and clinical data were downloaded from Xena [54,55]. Transcripts per million (TPM) matrix for protein-coding genes was constructed for each cancer type for the following analyses. For each sample, we used ESTIMATE [14] to calculate an immune score and a stromal score using the gene expression data. Then, for each of the 39 sets of biomarkers, we calculated the 48 scores for each sample. We performed 4 types of analyses to investigate the biomarker characteristics. First, we calculated the Spearman correlation coefficients (SCC) between any two scoring measurements, excluding the two categorical measurements EcoTyper and MFP. This resulted in a 46×46 correlation matrix that can be used to explore the correlations and potential duplicated information among these biomarkers and scores. Second, we calculated SCC between each score and TMB because previous studies had reported that samples with high TMB tended to respond well to ICB therapies. Third, we calculated SCC between each score and the immune and stromal scores, respectively, as both immune and stromal genes were reported to play roles in ICB response. Lastly, we investigated the functions of the biomarkers for their enriched pathways using the gene set enrichment analysis (GSEA) and the Reactome pathways [56].

### 2.7. ICB-Treated Data Collection and Benchmark

Data curation and preprocess. By a systematic search in the literature, we identified a total of 36 ICB-related studies with transcriptome data (Supplementary Table S1). Among these datasets, we excluded those that were generated using the NanoString panels [57,58] because such datasets had limited coverage of biomarker genes. We also excluded those that were under restricted control [32,59,60,61]. The remaining data were downloaded from public databases or the supplementary tables of the original publications. As a result, a total of 16 studies were retained, covering cancer types such as melanoma, urothelial cancer (UC), gastric cancer (GC), head and neck squamous cell carcinoma (HNSCC), clear cell renal cell carcinoma (ccRCC), non-small cell lung cancer (NSCLC), and glioblastoma (GBM). A total of 29 datasets were retrieved from these 16 studies and pre-processed uniformly to ensure coherence between the datasets. For each dataset, we kept only the transcripts of protein-coding genes and quantified their expression levels using TPM or other normalized metrics as expression units for the following analysis. All benchmark analyses were performed within each dataset and thus, the batch effect across different studies had limited impacts on our analyses. 

Definition of ICB-responder and ICB-nonresponder. Each of the original studies has its own way of defining ICB responders and ICB-nonresponders. Some studies used the Response Evaluation Criteria In Solid Tumors (RECIST) or immune-related RECIST (irRECIST) criteria [13,32,62,63,64,65]. Some studies used PFS for the stratification (e.g., lasting for 6 months after initiation of immunotherapy) [27,66,67]. Others used the combined information of the response status and survival information [38,63,68,69,70,71]. By reviewing the published strategies, we chose to use the combinatory strategy and uniformly assessed the clinical data and stratified patients as below:

ICB-responder: Patients with complete response (CR), partial response (PR), or stable disease (SD) as well as having PFS greater than 6 months.

ICB-nonresponder: Patients with progressive disease (PD) or SD but with PFS of less than 6 months.

After preprocessing, we kept 16 accessible studies including 1492 samples (Supplementary Table S2). Among them, 1337 samples were obtained before the treatment, which we collapsed into 15 datasets (Supplementary Table S3). Based on the clinical status (e.g., different biopsy time and treatment), we organized the 408 samples, including both pre-treatment and on-treatment samples from the same patients or samples with different treatments, as 14 datasets (Supplementary Table S4). In total, we obtained 29 datasets. All datasets were preprocessed to generate matched clinical data and gene expression matrix ready for further analyses. 

Evaluation using ICB-treated datasets. For each dataset with ICB-responders and ICB-nonresponders as defined above, we tested the association between each of the 48 scoring measurements with the ICB status using the one-sided Wilcoxon test (for the 46 continuous measurements) or Fisher’s Exact test (for the two categorial measurements). For datasets containing paired pre- and on-treatment samples derived from the same patients, we used the paired *t*-test to assess the changes in scores before and after ICB treatment. For the continuous measurement, we also calculated the AUC value using the R package *pROC*. For the datasets with the OS and PFS information, we categorized the patients based on the continuous scoring systems (using the mean values) or the prediction from the categorical scoring systems. Next, we conducted the Log-Rank test to compare the survival curves between the two groups of patients and generated the Kaplan-Meier (KM) plot. Finally, the Cox proportional hazards model and the forest plot of hazard ratio (HR) were employed to assess the impact of the biomarker on survival time. 

### 2.8. Statistical Analysis

All statistical analyses were performed in R (v4.1.3). Survival analyses including the Log-Rank test and Cox regression were conducted using the *survival* package. The KM plot and the forest plot were generated by using the *survminer* package.

### 2.9. Database and Web Server Construction

Most of the methods can be implemented using standalone tools, whereas some were made available as online web servers, such as TIDE [35], CIBERSORTx [49], and EcoTyper [45]. To facilitate easy access to the comprehensive datasets as well as the methods, we constructed the database named ICB-Portal (https://ngdc.cncb.ac.cn/icb (accessed on 1 July 2023)). The web server was hosted by a local machine with CentOS 7.9. The backend service was built with Java Spring Boot (https://spring.io/projects/spring-boot (accessed on 1 July 2023)) framework and MySQL v8.0 (https://www.mysql.com/ (accessed on 1 July 2023)) as the database engine. The user interface was constructed with React (https://reactjs.org/ (accessed on 1 July 2023)), Umi (https://umijs.org/ (accessed on 1 July 2023)), and Ant Design (https://ant.design/ (accessed on 1 July 2023)). Highcharts (https://www.highcharts.com/ (accessed on 1 July 2023)) and PlotyJS (https://plotly.com/javascript/ (accessed on 1 July 2023)) were used to provide interactive visual charts. Finally, the online analysis module was implemented by the Rserve (http://www.rforge.net/Rserve/ (accessed on 1 July 2023)) in the backend server.

## 3. Results

### 3.1. Classification of Transcriptomic Biomarkers of ICB Response

After literature search and curation, we collected a total of 39 sets of transcriptomic biomarkers and calculated 48 scoring measurements of ICB response based on three types of methods: the gene-set-like methods using the self-contained design, the gene-set-like methods using the competitive design, and the deconvolution-like methods (Table 1). Among the 48 scoring methods, 46 generated continuous measurements and two generated categorical measurements (i.e., the MFP and EcoTyper methods).

### 3.2. The Correlations and Patterns of Transcriptomic Biomarkers

According to the original studies, some scoring systems were initially developed to indicate beneficial ICB response while some for ICB resistance. Among the 48 scores, 33 were expected to be positively associated with the ICB treatment outcome, i.e., samples with a high score tended to have benefitting ICB response (hereafter referred as the positive markers), while the remaining 15 scores were negatively associated (negative markers).

**Figure 2:**
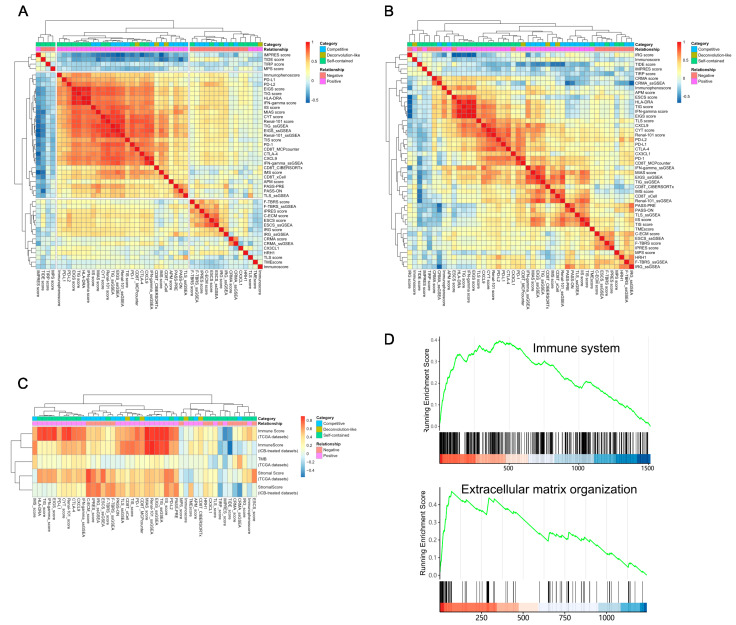
Characteristics of the transcriptomic biomarkers. (**A**) A heatmap showing the hierarchical clustering patterns for 46 biomarker score systems, excluding EcoTyper and MFP, which are categorical. For any pair of biomarkers, the correlation is calculated using the Spearman correlation coefficient using the approximately 8000 samples from 31 TCGA non-hematological cancer types. (**B**) A heatmap showing the hierarchical clustering patterns for 46 biomarker score systems based on the 16 ICB-treated datasets. (**C**) A heatmap displaying the association between biomarkers with TMB, the immune score, the stromal score, and the ESTIMATE score using TCGA datasets. (**D**) GSEA plots of the immune system pathway enriched in the positive biomarkers and the extracellular matrix organization pathway enriched in the negative biomarkers.

To verify these relationships, we first investigated the correlations among the 46 quantitative scores using the TCGA samples. We calculated all scores using the ~8000 TCGA samples from 31 non-hematological cancer types and constructed a 46 × 46 correlation matrix for the quantitative scores. As shown in Figure 2A, biomarkers that were expected with the same direction were clustered together roughly as expected, e.g., the positive markers formed a cluster and the negative markers formed another. The only exception was the IMPRES score, which had been reported as a positive biomarker but appeared to be closer to the negative marker cluster. The same trend was observed in the correlation analysis based on ICB-treated datasets (Figure 2B).

Next, we investigated the relationships between these biomarkers with three genomic and transcriptomic characteristics: TMB, the stromal score, and the immune score [14]. Overall, most of the biomarkers showed low correlations with TMB (mean: 0.11, range: −0.26~0.56) (Figure 2C). In addition, most of the positive biomarkers showed higher correlations with the immune score (mean: 0.60, range: −0.46~0.89, 30/31 = 96.78% > 0) than with the stromal score (mean: 0.36, range: −0.18~0.69, 27/31 = 87.10% > 0). In contrast, negative biomarkers showed higher correlations with the stromal score (mean: 0.34, range: −0.18~0.82, 12/15 = 80% > 0) than with the immune score (mean: 0.19, range: −0.41~0.46, 12/15 = 80% > 0) (Figure 2C).

To investigate the overlapping genes and the functional pathways that the component genes were enriched, we ranked the genes according to their frequency in either the positive biomarkers or the negative biomarkers. In this way, genes that were included in multiple biomarkers (thus, frequently identified as markers for ICB by multiple studies) received a high rank. As shown by the GSEA analyses, the genes from the positive biomarkers were significantly enriched in the immune system pathway (NES = 1.78, Benjamini-Hochberg Procedure adjusted *p*-value, or p_BH_ = 0.012), the adaptive immune system pathway (NES = 2.18, p_BH_ = 0.012) and the cytokine signaling in immune system pathway (NES = 1.64, p_BH_ = 0.012). Genes from the negative biomarkers were significantly enriched in the extracellular matrix organization pathway (NES = 4.75, p_BH_ = 0.012), signaling by *GPCR* pathway (NES = 2.15, p_BH_ = 0.012), and signaling by receptor tyrosine kinases pathway (NES = 2.38, p_BH_ = 0.012) (Figure 2D). These results implied that at least two different types of pathways were captured by the transcriptomic biomarkers that had been reported so far.

### 3.3. Benchmark of Transcriptomic Biomarkers for ICB Response Prediction

Overview of datasets: We next systematically quantified the predictive performance of these biomarkers using the ICB-treated datasets for ICB response. The 29 datasets after preprocessing included 1492 samples from five cancer types, i.e., melanoma (*n* = 535), NSCLC (*n* = 381), UC (*n* = 374), ccRCC (*n* = 157), and GC (*n* = 45). The overall objective response rate of total patients was 34.2%. Among the 29 datasets, 15 datasets were composed exclusively of samples taken before the ICB treatment, eight datasets were composed of paired pre- and on-treatment samples from the same patients, two datasets were designed to explore the difference between single and combined immunotherapy, and four datasets were designed to investigate the effects of previous exposure to anti-CTLA-4 before anti-PD1 treatment. In addition, there were 13 datasets with available OS information and 11 with PFS information. We named these datasets according to the cancer type, sample size, and the first author of original publication.

**Figure 3:**
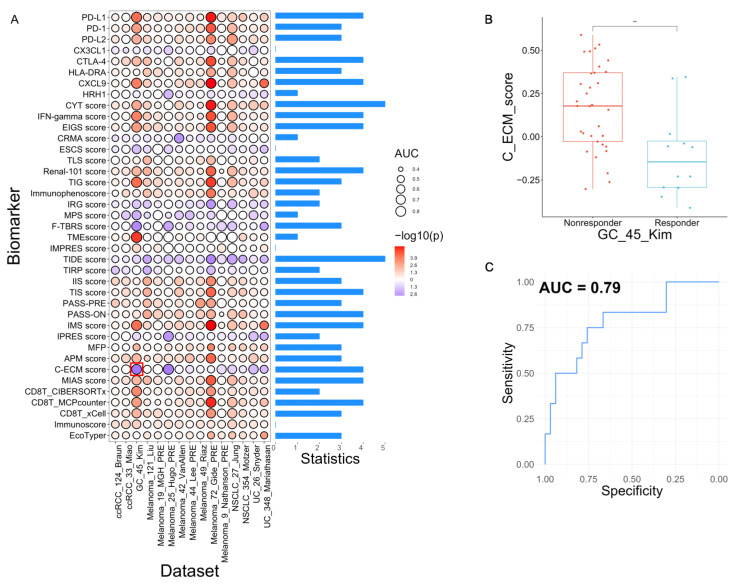
Benchmark results of 39 sets of transcriptomic biomarkers for ICB response. (**A**) A dot plot displaying the overall benchmark results of 39 biomarker score systems for ICB response. The color and size of the dots are proportional to the *p*-values and AUC values from the benchmark analyses of the corresponding biomarker (column) and the dataset (row), respectively. The bars on the right represent the counts of significant (*p* < 0.05) associations for the corresponding score systems. (**B**,**C**) Examples of benchmark results for C-ECM score (Biomarker) in GC_45_Kim (Dataset), which was highlighted by a red box in panel A. The dots in B represented patients (blue: responders, red: nonresponders). ** *p* < 0.01.

Benchmark using ICB responsive status: Using the 15 datasets that included only pre-treatment samples (*n* = 1337), we conducted one-sided statistical tests for each measurement (Wilcoxon test for 46 continuous measurements and Fisher’s Exact test for the two categorical measurements) to investigate their relationship with ICB response based on the pre-defined directions, i.e., positive or negative. As shown in Figure 3A, the TIDE and CYT scores showed the best performance which distinguished responders from nonresponders in 5 out of 15 datasets. Thirteen scores showed statistical significance in 4 out of 15 datasets and ten scores in 3 datasets. Lastly, four scores, which were CXCL1, ESCS, IMPRES, and CD8T_xCell, failed to distinguish the two groups of patients in all 15 datasets. We selected the C-ECM score in the GC_45_Kim dataset to demonstrate the results (Figure 3B,C) while all benchmark results are available via our website. Notably, for four of the benchmark datasets (i.e., ccRCC_124_Braun, ccRCC_32_Miao, Melanoma_19_MGH_PRE, and Melanoma_19_Nathanson_PRE), all biomarkers failed to distinguish the patient groups (Figure 3A), likely due to the tumor heterogeneity and the small sample sizes [38,66,69,72]. In general, the cancer-specific biomarkers have better performance for the same cancer as they derive from than other cancers. For example, twelve of fifteen melanoma-specific biomarkers have the top significant *p*-values in the melanoma datasets except for MPS, Immunoscore and IMPRES scores. The TIDE score showed significant predictive power in five datasets, four of which were the same as the cancer types used to construct the TIDE (Melanoma, NSCLC). However, the CYT score is a general biomarker which showed significant predictive power in five datasets with more diverse cancer types (Melanoma, GC, NSCLC, UC).

**Figure 4:**
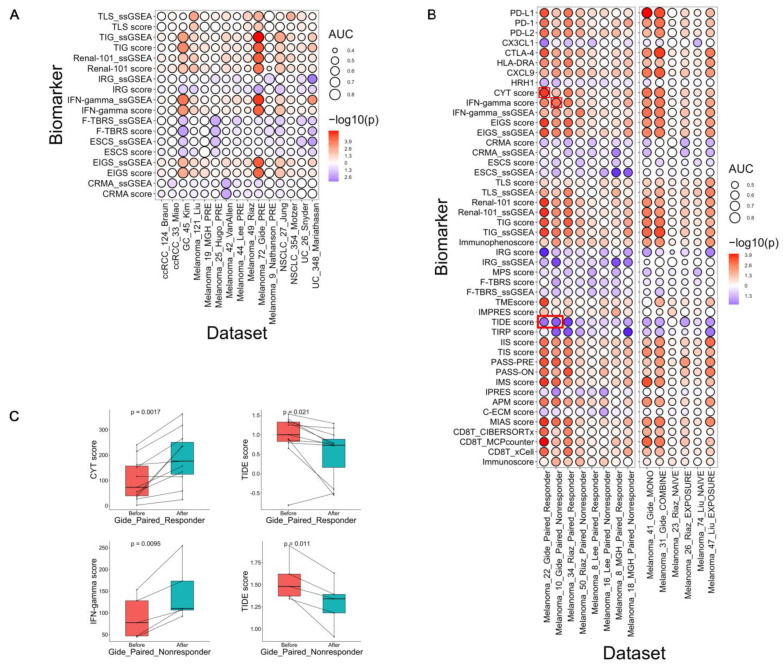
Comparison of different computational methods, biopsy times, and treatments. (**A**) Benchmark results using different calculation methods for ICB response prediction. The color of each dot is proportional to the *p* value and the size is proportional to the AUC value from the benchmark. (**B**) Benchmark results for pre- and on-treatment samples (left) and for different treatments (right) (see main text for details). The analyses were conducted using 46 continuous score systems. (**C**) Examples (highlighted by a red box in panel B) of biomarker score changes before and after ICB treatment (paired *t*-test) in responders and nonresponders.

Comparison of computational methods: For the nine biomarkers that were originally calculated using the average or sum of expression of component genes [23,24,25,26,27,30,32], we further applied ssGSEA using the same genes to calculate a new score. This allowed us to investigate the impact of different computation methods. As shown in Figure 4A, the results generally showed that, for the same gene signature, four new score measurements derived from ssGSEA, including TLS_ssGSEA, TIG_ssGSEA, Renal-101_ssGSEA, and ESCS_ssGSEA, were more predictive of the ICB response than the original score measurements derived from average or sum. In addition, three new scores (EIGS_ssGSEA, IFN-gamma_ssGSEA, and CRMA_ssGSEA) showed comparable performance with the original score and two new scores (IRG_ssGSEA and F-TBRS_ssGSEA) showed poorer performance than the original scores (Figure 4A). Taken together, ssGSEA was in general superior to naïve calculation methods of gene signatures. A possible explanation was that ssGSEA assessed the relative expression changes in a set of genes as compared with the rest of genes in the transcriptome and thus, was robust to confounding factors such as sample preparation and technical platforms.

Benchmark using different biopsy groups: Four studies including 83 patients had paired pre- and on-treatment samples (166 transcriptome) regardless of the type of treatments (e.g., anti-CTLA-1 or anti-PD-1). For these datasets, we stratified the patients as responders and nonresponders and investigated the changes in the 46 continuous biomarker scores upon ICB treatments [38,62,63,65,68]. In total, more biomarkers were significantly changed in responders than in nonresponders (Figure 4B) and positive scores tended to increase whereas negative scores decrease upon treatment. Specifically, for responders in each dataset (Gide: *n* = 22, Riaz: *n* = 34, Lee: *n* = 8, MGH: *n* = 8), 32 scores showed significant changes before and on ICB treatment (paired *t*-test, *p* < 0.05), including 25 scores upregulated and 7 downregulated (Figure 4B, Supplementary Table S5). In addition, for nonresponders in each dataset (Gide: *n* = 10, Riaz: *n* = 50, Lee: *n* = 16, MGH: *n* = 18), 21 scores showed significant changes (paired *t*-test, *p* < 0.05; 18 upregulated and 3 downregulated) (Figure 4B, Supplementary Table S5). As reported in previous studies, many immune genes were found activated during ICB treatment [63], likely because of the activated immune pathways during treatment. Thus, it is expected that biomarkers consisting of immune related genes would increase upon ICB treatments. In our results, among the upregulated scores, regardless of responders and nonresponders, the majority of them were calculated using immune related genes, consistent with previous reports. For example, the CYT and IFN-gamma scores showed significant upregulation after ICB treatment in both responders and nonresponders. (Figure 4C). In contrast, the scores that decreased were all from the negative sets including those related to TME (such as ESCS and TMEscore) and immune resistance program (such as TIRP, CRMA, and IRG).

Benchmark using different treatment groups: We also evaluated the performance of the scores using data from three studies that collected samples treated with different treatments. The Riaz and Liu studies compared samples naïve to anti-CTLA-4 before anti-PD-1 treatment (hereafter named naïve samples, Riaz: *n* = 23, Liu: *n* = 74) with samples that were derived from exposure to anti-CTLA-4 before anti-PD-1 treatment (exposed, Riaz: *n* = 26, Liu: *n* = 47). Specifically, 29 scores were significantly higher in responder than in nonresponder using the exposed samples (one-sided Wilcoxon test, *p* < 0.05), but only one showed significant difference in the naïve sample group. One possible explanation is that the higher expression of various immune-related pathways distinguished responders from nonresponders in previous exposure to anti-CTLA-4 patients but not anti-CTLA-4-naïve patients [62]. The Gide study compared samples treated with anti-PD-1 monotherapy (mono, *n* = 41) and samples treated with combined therapy with both anti-PD-1 and anti-CTLA-4 (combined, *n* = 31) [68]. We found 29 scores significantly higher in responder than in nonresponder in the group treated with the combined therapy, whereas 26 scores were higher in responder than in nonresponder in the mono therapy group (one-sided Wilcoxon test *p* < 0.05) (Figure 4B, Supplementary Table S5). Taken together, these results showed that these biomarkers are also limited in discriminating responders from nonresponders in samples treated with monoclonal antibodies.

### 3.4. Exploration of Biomarkers for Their Prognostic Capability

**Figure 5:**
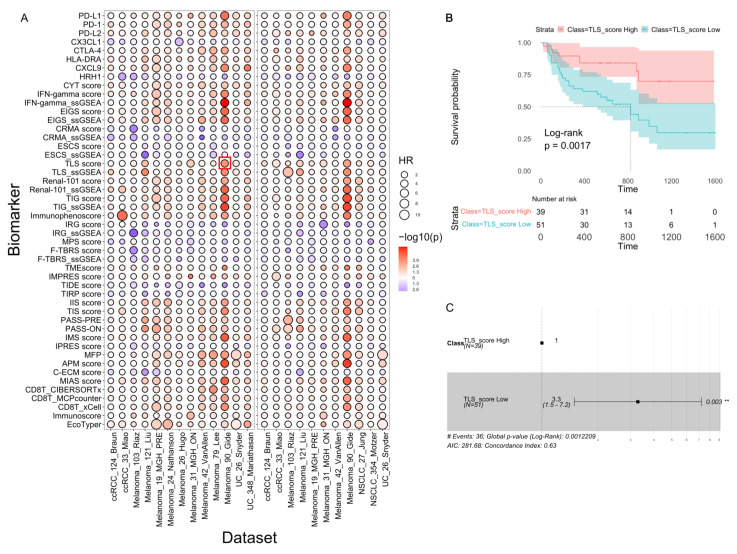
Benchmark of the prognostic capability for the 48 score systems using patient OS and PFS. (**A**) The benchmark results of the biomarkers for OS (left box) and PFS (right box). The color of each dot is proportional to the *p* value and the size is proportional to the HR value derived from the benchmark analysis of the corresponding biomarker and the dataset. (**B**,**C**) Examples (highlighted by a red box in panel A) of the detailed benchmark results using the TLS score in the Melanoma_90_Gide dataset for OS, including the KM plot (**B**) and the forest plot (**C**). ** *p* < 0.01.

We evaluated the prognostic capability of the 48 scores using both the ICB-treated datasets and the TCGA datasets (31 cancer types). In the ICB-treated datasets (Figure 5A), PASS-ON and EIGS_ssGSEA showed the best performance for both OS and PFS. Figure 5B,C showed an example of the KM-plot and forest plot using the TLS score in the Gide dataset. Interestingly, there were a number of biomarkers that have great predictive power for ICB response but poor prognostic capability for OS and PFS, and vice versa. For example, TIDE was the best predictive biomarker for ICB response (Figure 3A) but failed to show significant prognostic value in most datasets (Figure 5A). This is partially because TIDE mainly contains genes interacting with cytotoxic T lymphocytes (CTL) and these genes may not be directly associated with survival [35].

In the TCGA datasets, F-TBRS score achieved the best performance (Supplementary Figure S1). Note that we included only solid tumor samples in TCGA and these samples may have different levels of fibroblasts and TME. Moreover, as expected, the dataset of skin cutaneous melanoma (SKCM) has the highest number of biomarkers with significant predictive power for OS. This is expected because most biomarkers were originally developed in melanoma cohorts.

### 3.5. Web Server Construction

To facilitate access of the curated resources and the benchmark results, we developed the database ICB-Portal, available at https://ngdc.cncb.ac.cn/icb (accessed on 1 July 2023). All 29 datasets, 48 score systems, corresponding benchmark results, as well as an online implementation of these scores were made available in ICB-Portal, representing the most comprehensive resource currently in the field of transcriptomic ICB [73,74] (Supplementary Figure S2). We provided various interactive and friendly ways to explore the detailed information of the datasets, biomarkers, and the benchmark results (Supplementary Figure S2A,B). The online analysis platform provides a one-stop solution for researchers to test their custom biomarkers using the 29 datasets without extra manual curation and pre-processing (Supplementary Figure S2C). Specifically, the custom biomarker entered by the user can be a single gene or a gene set. We provide three calculations when a gene set is submitted, including ssGSEA, average, and sum of all genes. A job ID is generated for the accessibility of corresponding results after the submission, and a job is completed within a few minutes in general. Altogether, we believe this module accelerates the discovery of novel therapeutic targets and potential predictors for immunotherapy.

## 4. Discussion

In this study, we collected 29 ICB-treated datasets and 48 scoring systems from 39 sets of transcriptome biomarkers and conducted a comprehensive benchmark of these biomarkers. To the best of our knowledge, these datasets represented the most comprehensive resource that are publicly available, covering different cancer types, biopsy times, and treatment scenarios. In our benchmark results, most biomarkers have shown poor performance in terms of stability and robustness across different datasets. Additionally, we validated the prognostic power of these biomarkers in ICB-treated datasets and TCGA datasets. Lastly, these curated resources and analysis results were compiled into a website for convenient browsing and an online benchmark platform was provided for users to test their custom biomarkers in the 29 datasets for ICB response and association with clinical outcome. We expect that our webserver, the ICB-Portal, can serve as a comprehensive resource for ICB-related transcriptomics research and a useful tool for identifying or validating novel biomarkers.

Our work has the following limitations. First, the biomarkers investigated in this study are primarily based on transcriptomic data. However, there are also genomic features that can serve as biomarkers for ICB response, such as TMB [6,10,59], mismatch-repaid deficiency [11], recurrent somatic mutations [12], and aneuploidy [8]. Recent studies have shown that integration of multi-omics data and demographic and clinical data can be a more optimized manner to identify critical determinants for immune response [5,75]. Second, the development of new methods and technologies also facilitates the improvement the biomarker, such as cell type-specific ligand-receptor interactions [76] and the spatial relationships of cellular components [77]. Third, in addition to these biomarkers established using tissue samples, several biomarkers have been identified in peripheral blood [78,79,80], which were more non-invasive and clinically meaningful. Finally, most of the publicly available ICB-treated datasets tend to have small sample sizes except for a few large cohorts, which may make it difficult to reach statistical significance when benchmarking the biomarker. In the future, when large-scale transcriptome data are released by clinical trial studies, more comprehensive benchmark can be conducted to warrant the results and to develop novel biomarkers.

Despite these limitations, our labor-intensive curation and systematic benchmark analysis can provide important insights into the understanding of ICB response. By focusing on the transcriptomic biomarkers for ICB response, our analyses highlighted the urgent need to develop more robust and stable biomarkers across different cancer types, biopsy times and treatment scenarios. We expect the ICB-Portal will serve as a valuable reference resource and validation tool for ICB-related research.

## 5. Conclusions

We curated 29 ICB-treated datasets covering different cancer types, biopsy times, and treatment scenarios, which represented the most comprehensive resource so far and 39 sets of transcriptomic biomarkers involving 48 scoring systems. Then, we categorized these methods into three groups: the gene-set-like group with self-contained design, the gene-set-like group with competitive design, and the deconvolution-like group. Moreover, a comprehensive benchmark of the scoring systems from various aspects were conducted. Finally, we constructed an online service, ICB-portal, which provides a one-stop shop for ICB-related research.

## Figures and Tables

**Table 1:** Summary of 48 scores in this study.

Biomarker Score	Category	Description	Tumor Type	Effect	Antibody	Ref.
*PD-L1*	Self-contained	Expr. of *PD-L1*	Multiple	Pos.	anti-PD-1 anti-PD-L1	[15,16]
*PD-1*	Self-contained	Expr. of *PD-1*	Multiple	Pos.	anti-PD-1	[17]
*PD-L2*	Self-contained	Expr. of *PD-L2*	Multiple	Pos.	anti-PD-1	[18]
*CX3CL1*	Self-contained	Expr. of *CX3CL1*	Multiple	Neg.	anti-PD-L1	[15]
*CTLA-4*	Self-contained	Expr. of *CTLA4*	Multiple	Pos.	anti-PD-L1	[15]
*HLA-DRA*	Self-contained	Expr. of *HLA-DRA*	Melanoma	Pos.	anti-PD-1 anti-PD-L1	[19]
*CXCL9*	Self-contained	Expr. of *CXCL9*	Melanoma	Pos.	anti-PD-L1	[20]
*HRH1*	Self-contained	Expr. of *HRH1*	Melanoma Lung cancer	Neg.	anti-PD-1 anti-PD-L1 anti-CTLA-4	[21]
CYT score	Self-contained	Avg.expr. of *GZMA* and *PRF1*	Multiple	Pos.	anti-CTLA-4 anti-PD-1	[22]
IFN-gamma score	Self-contained	Avg.expr. of 6 genes	Multiple	Pos.	anti-PD-1	[23]
EIGS score	Self-contained	Avg.expr. of 18 genes	Multiple	Pos.	anti-PD-1	[23]
CRMA score	Self-contained	Avg.expr. of 8 genes	Melanoma	Neg.	anti-CTLA-4	[24]
ESCS score	Self-contained	Avg.expr. of 8 genes	UC	Neg.	anti-PD-1	[25]
TLS score	Self-contained	Avg.expr. of 9 genes	Melanoma	Pos.	anti-PD-1 anti-CTLA-4	[26]
Renal-101 score	Self-contained	Avg.expr. of 26 genes	RCC	Pos.	anti-PD-1 anti-PD-L1	[27]
TIG score	Self-contained	Weighted sum of 18 genes	Multiple	Pos.	anti-PD-1	[23,28]
Immunophenoscore	Self-contained	Weighted sum of 162 genes	Multiple	Pos.	anti-CTLA-4 anti-PD-1	[29]
IRG score	Self-contained	Weighted sum of 11 genes	Cervical Cancer	Neg.	anti-PD-1 anti-PD-L1 anti-CTLA-4	[30]
MPS score	Self-contained	Weighted sum of 45 genes	Melanoma	Neg.	anti-PD-1 anti-CTLA-4	[31]
F-TBRS score	Self-contained	PCA using 19 genes	Multiple	Neg.	anti-PD-L1	[32]
TMEscore	Self-contained	PCA using 2 GSs	Gastric Cancer	Pos.	anti-PD-1 anti-PD-L1 anti-CTLA-4	[33]
IMPRES score	Self-contained	15 pairwise immune checkpoint genes	Melanoma	Pos.	anti-PD-1 anti-CTLA-4	[34]
TIDE score	Self-contained	Modeling 2 primary mechanisms of tumor immune evasion	Melanoma NSCLC	Neg.	anti-PD-1 anti-CTLA-4	[35]
TIRP score	Self-contained	OE of immune resistance program	Melanoma	Neg.	anti-PD-1	[36]
IIS score	Competitive	Sum NESs of 26 related GSs	ccRCC	Pos.	anti-PD-1	[37]
TIS score	Competitive	Sum NESs of 8 related GSs	ccRCC	Pos.	anti-PD-1	[37]
PASS-PRE	Competitive	Weighted sum of NESs of 15 GSs	Melanoma	Pos.	anti-PD-1	[38]
PASS-ON	Competitive	Weighted sum of NESs of 15 GSs	Melanoma	Pos.	anti-PD-1	[38]
IMS score	Competitive	Weighted sum of NESs of 27 GSs	Gastric Cancer	Pos.	anti-PD-1 anti-PD-L1	[39]
IPRES score	Competitive	Mean NESs of 26 GSs	Multiple	Neg.	anti-PD-1	[13]
MFP	Competitive	Classification of samples based on NES	Multiple	Pos.	anti-PD-1 anti-PD-L1 anti-CTLA-4	[40]
APM score	Competitive	NES of antigen presentation related GS	ccRCC	Pos.	anti-PD-1	[37]
C-ECM score	Competitive	NES of ECM-related GS	Multiple	Neg.	anti-PD-1	[41]
MIAS score	Competitive	NES of MHC I related GS	Melanoma	Pos.	anti-PD-1	[42]
IFN-gamma_ssGSEA	Competitive	NES of related genes	Multiple	Pos.	anti-PD-1	[23]
EIGS_ssGSEA	Competitive	NES of corresponding GS	Multiple	Pos.	anti-PD-1	[23]
TIG_ssGSEA	Competitive	NES of corresponding GS	Multiple	Pos.	anti-PD-1	[23]
CRMA_ssGSEA	Competitive	NES of corresponding GS	Melanoma	Neg.	anti-CTLA-4	[24]
ESCS_ssGSEA	Competitive	NES of corresponding GS	UC	Neg.	anti-PD-1	[25]
F-TBRS_ssGSEA	Competitive	NES of corresponding GS	Multiple	Neg.	anti-PD-L1	[32]
IRG_ssGSEA	Competitive	NES of corresponding GS	Cervical Cancer	Neg.	anti-PD-1 anti-PD-L1 anti-CTLA-4	[30]
TLS_ssGSEA	Competitive	NES of corresponding GS	Melanoma	Pos.	anti-PD-1 anti-CTLA-4	[26]
Renal-101_ssGSEA	Competitive	NES of corresponding GS	RCC	Pos.	anti-PD-1 anti-PD-L1	[27]
CD8T_CIBERSORTx	Deconvolution-like	Tumor infiltration of CD8 T cells	Multiple	Pos.	anti-PD-1	[43]
CD8T_MCPcounter	Deconvolution-like	Tumor infiltration of CD8 T cells	Multiple	Pos.	anti-PD-1	[43]
CD8T_xCell	Deconvolution-like	Tumor infiltration of CD8 T cells	Multiple	Pos.	anti-PD-1	[43]
Immunoscore	Deconvolution-like	Weighted sum of the fraction levels of 8 cell types	Melanoma	Pos.	anti-PD-1	[44]
EcoTyper	Deconvolution-like	Carcinoma ecotypes	Multiple	Pos.	anti-PD-1 anti-PD-L1 anti-CTLA-4	[45]

Abbreviations: PCA: principal component analysis; OE: overall expression; ECM: extracellular matrix; IRG score: immune-related risk score; IRG_ssGSEA: immune-related risk ssGSEA; EIGS score: expanded immune gene signature score; EIGS_ssGSEA: expanded immune gene signature ssGSEA; ESCS score: EMT stroma core signature score; ESCS_ssGSEA: ssGSEA of EMT stroma core signature; Renal-101 score: Renal-101 immune signature score; Renal-101_ssGSEA: ssGSEA of Renal-101 immune signature; TIG score: T cell-inflamed GEP score; TIG_ssGSEA: ssGSEA of T cell-inflamed GEP; TIRP score: the immune resistance program score; NSCLC: non-small cell lung cancer; ccRCC: clear cell renal cell carcinoma; RCC: renal cell carcinoma; UC: urothelial cancer; GS: gene set; Pos.: positive; Neg.: negative; Expr.: expression; Avg.expr.: average expression.

## Data Availability

All processed data, benchmark results and source code can be downloaded in the ICB-Portal (https://ngdc.cncb.ac.cn/icb/resources (accessed on 1 July 2023)). There are no limitations or restrictions on the data usage. All the curated datasets were derived from public studies.

## Supplementary Materials

The following supporting information can be downloaded at: https://www.mdpi.com/article/10.3390/cancers15164094/s1, Supplementary Figure S1: Evaluation of the prognostic capability for the 48 score systems using patient OS across TCGA datasets. Supplementary Figure S2: The web interface of ICB-Portal. (A) A screenshot of the ICB Biomarker Atlas. (B) An example of the dataset page. C. The interface of the online analysis platform. Supplementary Table S1: A total manually curated list of 37 existing ICB-treated studies with transcriptome data derived from manually literature curation. The rows marked in green are the datasets that eventually be used for the following analysis. Supplementary Table S2: A total of 16 public datasets were obtained after a uniformly preprocessing, each with the matched clinical data and standardized expression matrix. Supplementary Table S3: Summary of 15 datasets containing purely pre-treatment samples used for the benchmarking of predictive performance for ICB response. Supplementary Table S4: Summary of 8 datasets composed of paired pre-and on-treatment samples from the same patient group and 6 datasets composed of different treatment samples. Supplementary Table S5: Summary of all benchmark results for the 48 score systems.

## References

[1] Schachter J., Ribas A., Long G.V., Arance A., Grob J.-J., Mortier L., Daud A., Carlino M.S., McNeil C., Lotem M. (2017). Pembrolizumab versus ipilimumab for advanced melanoma: Final overall survival results of a multicentre, randomised, open-label phase 3 study (KEYNOTE-006). Lancet.

[2] Herbst R.S., Baas P., Kim D.-W., Felip E., Pérez-Gracia J.L., Han J.-Y., Molina J., Kim J.-H., Arvis C.D., Ahn M.-J. (2016). Pembrolizumab versus docetaxel for previously treated, PD-L1-positive, advanced non-small-cell lung cancer (KEYNOTE-010): A randomised controlled trial. Lancet.

[3] Xing P., Zhang F., Wang G., Xu Y., Li C., Wang S., Guo Y., Cai S., Wang Y., Li J. (2019). Incidence rates of immune-related adverse events and their correlation with response in advanced solid tumours treated with NIVO or NIVO+IPI: A systematic review and meta-analysis. J. Immunother. Cancer.

[4] Postow M.A., Sidlow R., Hellmann M.D. (2018). Immune-Related Adverse Events Associated with Immune Checkpoint Blockade. N. Engl. J. Med..

[5] Chowell D., Yoo S.K., Valero C., Pastore A., Krishna C., Lee M., Hoen D., Shi H., Kelly D.W., Patel N. (2021). Improved prediction of immune checkpoint blockade efficacy across multiple cancer types. Nat. Biotechnol..

[6] Samstein R.M., Lee C.H., Shoushtari A.N., Hellmann M.D., Shen R., Janjigian Y.Y., Barron D.A., Zehir A., Jordan E.J., Omuro A. (2019). Tumor mutational load predicts survival after immunotherapy across multiple cancer types. Nat. Genet..

[7] Keenan T.E., Burke K.P., Van Allen E.M. (2019). Genomic correlates of response to immune checkpoint blockade. Nat. Med..

[8] Davoli T., Uno H., Wooten E.C., Elledge S.J. (2017). Tumor aneuploidy correlates with markers of immune evasion and with reduced response to immunotherapy. Science.

[9] Miao D., Margolis C.A., Vokes N.I., Liu D., Taylor-Weiner A., Wankowicz S.M., Adeegbe D., Keliher D., Schilling B., Tracy A. (2018). Genomic correlates of response to immune checkpoint blockade in microsatellite-stable solid tumors. Nat. Genet..

[10] Snyder A., Makarov V., Merghoub T., Yuan J., Zaretsky J.M., Desrichard A., Walsh L.A., Postow M.A., Wong P., Ho T.S. (2014). Genetic basis for clinical response to CTLA-4 blockade in melanoma. N. Engl. J. Med..

[11] Le D.T., Uram J.N., Wang H., Bartlett B.R., Kemberling H., Eyring A.D., Skora A.D., Luber B.S., Azad N.S., Laheru D. (2015). PD-1 Blockade in Tumors with Mismatch-Repair Deficiency. N. Engl. J. Med..

[12] Gajic Z.Z., Deshpande A., Legut M., Imielinski M., Sanjana N.E. (2022). Recurrent somatic mutations as predictors of immunotherapy response. Nat. Commun..

[13] Hugo W., Zaretsky J.M., Sun L., Song C., Moreno B.H., Hu-Lieskovan S., Berent-Maoz B., Pang J., Chmielowski B., Cherry G. (2016). Genomic and Transcriptomic Features of Response to Anti-PD-1 Therapy in Metastatic Melanoma. Cell.

[14] Yoshihara K., Shahmoradgoli M., Martinez E., Vegesna R., Kim H., Torres-Garcia W., Trevino V., Shen H., Laird P.W., Levine D.A. (2013). Inferring tumour purity and stromal and immune cell admixture from expression data. Nat. Commun..

[15] Herbst R.S., Soria J.C., Kowanetz M., Fine G.D., Hamid O., Gordon M.S., Sosman J.A., McDermott D.F., Powderly J.D., Gettinger S.N. (2014). Predictive correlates of response to the anti-PD-L1 antibody MPDL3280A in cancer patients. Nature.

[16] Patel S.P., Kurzrock R. (2015). PD-L1 Expression as a Predictive Biomarker in Cancer Immunotherapy. Mol. Cancer Ther..

[17] Taube J.M., Klein A., Brahmer J.R., Xu H., Pan X., Kim J.H., Chen L., Pardoll D.M., Topalian S.L., Anders R.A. (2014). Association of PD-1, PD-1 ligands, and other features of the tumor immune microenvironment with response to anti-PD-1 therapy. Clin. Cancer Res..

[18] Yearley J.H., Gibson C., Yu N., Moon C., Murphy E., Juco J., Lunceford J., Cheng J., Chow L.Q.M., Seiwert T.Y. (2017). PD-L2 Expression in Human Tumors: Relevance to Anti-PD-1 Therapy in Cancer. Clin. Cancer Res..

[19] Johnson D.B., Estrada M.V., Salgado R., Sanchez V., Doxie D.B., Opalenik S.R., Vilgelm A.E., Feld E., Johnson A.S., Greenplate A.R. (2016). Melanoma-specific MHC-II expression represents a tumour-autonomous phenotype and predicts response to anti-PD-1/PD-L1 therapy. Nat. Commun..

[20] Qu Y., Wen J., Thomas G., Yang W., Prior W., He W., Sundar P., Wang X., Potluri S., Salek-Ardakani S. (2020). Baseline Frequency of Inflammatory Cxcl9-Expressing Tumor-Associated Macrophages Predicts Response to Avelumab Treatment. Cell Rep..

[21] Li H., Xiao Y., Li Q., Yao J., Yuan X., Zhang Y., Yin X., Saito Y., Fan H., Li P. (2022). The allergy mediator histamine confers resistance to immunotherapy in cancer patients via activation of the macrophage histamine receptor H1. Cancer Cell.

[22] Rooney M.S., Shukla S.A., Wu C.J., Getz G., Hacohen N. (2015). Molecular and genetic properties of tumors associated with local immune cytolytic activity. Cell.

[23] Ayers M., Lunceford J., Nebozhyn M., Murphy E., Loboda A., Kaufman D.R., Albright A., Cheng J.D., Kang S.P., Shankaran V. (2017). IFN-gamma-related mRNA profile predicts clinical response to PD-1 blockade. J. Clin. Investig..

[24] Shukla S.A., Bachireddy P., Schilling B., Galonska C., Zhan Q., Bango C., Langer R., Lee P.C., Gusenleitner D., Keskin D.B. (2018). Cancer-Germline Antigen Expression Discriminates Clinical Outcome to CTLA-4 Blockade. Cell.

[25] Wang L., Saci A., Szabo P.M., Chasalow S.D., Castillo-Martin M., Domingo-Domenech J., Siefker-Radtke A., Sharma P., Sfakianos J.P., Gong Y. (2018). EMT- and stroma-related gene expression and resistance to PD-1 blockade in urothelial cancer. Nat. Commun..

[26] Cabrita R., Lauss M., Sanna A., Donia M., Skaarup Larsen M., Mitra S., Johansson I., Phung B., Harbst K., Vallon-Christersson J. (2020). Tertiary lymphoid structures improve immunotherapy and survival in melanoma. Nature.

[27] Motzer R.J., Robbins P.B., Powles T., Albiges L., Haanen J.B., Larkin J., Mu X.J., Ching K.A., Uemura M., Pal S.K. (2020). Avelumab plus axitinib versus sunitinib in advanced renal cell carcinoma: Biomarker analysis of the phase 3 JAVELIN Renal 101 trial. Nat. Med..

[28] Cristescu R., Mogg R., Ayers M., Albright A., Murphy E., Yearley J., Sher X., Liu X.Q., Lu H., Nebozhyn M. (2018). Pan-tumor genomic biomarkers for PD-1 checkpoint blockade-based immunotherapy. Science.

[29] Charoentong P., Finotello F., Angelova M., Mayer C., Efremova M., Rieder D., Hackl H., Trajanoski Z. (2017). Pan-cancer Immunogenomic Analyses Reveal Genotype-Immunophenotype Relationships and Predictors of Response to Checkpoint Blockade. Cell Rep..

[30] Yang S., Wu Y., Deng Y., Zhou L., Yang P., Zheng Y., Zhang D., Zhai Z., Li N., Hao Q. (2019). Identification of a prognostic immune signature for cervical cancer to predict survival and response to immune checkpoint inhibitors. Oncoimmunology.

[31] Perez-Guijarro E., Yang H.H., Araya R.E., El Meskini R., Michael H.T., Vodnala S.K., Marie K.L., Smith C., Chin S., Lam K.C. (2020). Multimodel preclinical platform predicts clinical response of melanoma to immunotherapy. Nat. Med..

[32] Mariathasan S., Turley S.J., Nickles D., Castiglioni A., Yuen K., Wang Y., Kadel E.E., Koeppen H., Astarita J.L., Cubas R. (2018). TGFbeta attenuates tumour response to PD-L1 blockade by contributing to exclusion of T cells. Nature.

[33] Zeng D., Li M., Zhou R., Zhang J., Sun H., Shi M., Bin J., Liao Y., Rao J., Liao W. (2019). Tumor Microenvironment Characterization in Gastric Cancer Identifies Prognostic and Immunotherapeutically Relevant Gene Signatures. Cancer Immunol. Res..

[34] Auslander N., Zhang G., Lee J.S., Frederick D.T., Miao B., Moll T., Tian T., Wei Z., Madan S., Sullivan R.J. (2018). Robust prediction of response to immune checkpoint blockade therapy in metastatic melanoma. Nat. Med..

[35] Jiang P., Gu S., Pan D., Fu J., Sahu A., Hu X., Li Z., Traugh N., Bu X., Li B. (2018). Signatures of T cell dysfunction and exclusion predict cancer immunotherapy response. Nat. Med..

[36] Jerby-Arnon L., Shah P., Cuoco M.S., Rodman C., Su M.J., Melms J.C., Leeson R., Kanodia A., Mei S., Lin J.R. (2018). A Cancer Cell Program Promotes T Cell Exclusion and Resistance to Checkpoint Blockade. Cell.

[37] Senbabaoglu Y., Gejman R.S., Winer A.G., Liu M., Van Allen E.M., de Velasco G., Miao D., Ostrovnaya I., Drill E., Luna A. (2016). Tumor immune microenvironment characterization in clear cell renal cell carcinoma identifies prognostic and immunotherapeutically relevant messenger RNA signatures. Genome Biol..

[38] Du K., Wei S., Wei Z., Frederick D.T., Miao B., Moll T., Tian T., Sugarman E., Gabrilovich D.I., Sullivan R.J. (2021). Pathway signatures derived from on-treatment tumor specimens predict response to anti-PD1 blockade in metastatic melanoma. Nat. Commun..

[39] Lin Y., Pan X., Zhao L., Yang C., Zhang Z., Wang B., Gao Z., Jiang K., Ye Y., Wang S. (2021). Immune cell infiltration signatures identified molecular subtypes and underlying mechanisms in gastric cancer. NPJ Genom. Med..

[40] Bagaev A., Kotlov N., Nomie K., Svekolkin V., Gafurov A., Isaeva O., Osokin N., Kozlov I., Frenkel F., Gancharova O. (2021). Conserved pan-cancer microenvironment subtypes predict response to immunotherapy. Cancer Cell.

[41] Chakravarthy A., Khan L., Bensler N.P., Bose P., De Carvalho D.D. (2018). TGF-beta-associated extracellular matrix genes link cancer-associated fibroblasts to immune evasion and immunotherapy failure. Nat. Commun..

[42] Wu C.C., Wang Y.A., Livingston J.A., Zhang J., Futreal P.A. (2022). Prediction of biomarkers and therapeutic combinations for anti-PD-1 immunotherapy using the global gene network association. Nat. Commun..

[43] Tumeh P.C., Harview C.L., Yearley J.H., Shintaku I.P., Taylor E.J., Robert L., Chmielowski B., Spasic M., Henry G., Ciobanu V. (2014). PD-1 blockade induces responses by inhibiting adaptive immune resistance. Nature.

[44] Nie R.C., Yuan S.Q., Wang Y., Chen Y.B., Cai Y.Y., Chen S., Li S.M., Zhou J., Chen G.M., Luo T.Q. (2019). Robust immunoscore model to predict the response to anti-PD1 therapy in melanoma. Aging.

[45] Luca B.A., Steen C.B., Matusiak M., Azizi A., Varma S., Zhu C., Przybyl J., Espin-Perez A., Diehn M., Alizadeh A.A. (2021). Atlas of clinically distinct cell states and ecosystems across human solid tumors. Cell.

[46] Chen Z., Zhou L., Liu L., Hou Y., Xiong M., Yang Y., Hu J., Chen K. (2020). Single-cell RNA sequencing highlights the role of inflammatory cancer-associated fibroblasts in bladder urothelial carcinoma. Nat. Commun..

[47] Aran D., Hu Z., Butte A.J. (2017). xCell: Digitally portraying the tissue cellular heterogeneity landscape. Genome Biol..

[48] Becht E., Giraldo N.A., Lacroix L., Buttard B., Elarouci N., Petitprez F., Selves J., Laurent-Puig P., Sautes-Fridman C., Fridman W.H. (2016). Estimating the population abundance of tissue-infiltrating immune and stromal cell populations using gene expression. Genome Biol..

[49] Newman A.M., Steen C.B., Liu C.L., Gentles A.J., Chaudhuri A.A., Scherer F., Khodadoust M.S., Esfahani M.S., Luca B.A., Steiner D. (2019). Determining cell type abundance and expression from bulk tissues with digital cytometry. Nat. Biotechnol..

[50] Newman A.M., Liu C.L., Green M.R., Gentles A.J., Feng W., Xu Y., Hoang C.D., Diehn M., Alizadeh A.A. (2015). Robust enumeration of cell subsets from tissue expression profiles. Nat. Methods.

[51] Grasso C.S., Tsoi J., Onyshchenko M., Abril-Rodriguez G., Ross-Macdonald P., Wind-Rotolo M., Champhekar A., Medina E., Torrejon D.Y., Shin D.S. (2020). Conserved Interferon-gamma Signaling Drives Clinical Response to Immune Checkpoint Blockade Therapy in Melanoma. Cancer Cell.

[52] Colaprico A., Silva T.C., Olsen C., Garofano L., Cava C., Garolini D., Sabedot T.S., Malta T.M., Pagnotta S.M., Castiglioni I. (2016). TCGAbiolinks: An R/Bioconductor package for integrative analysis of TCGA data. Nucleic Acids Res..

[53] Mayakonda A., Lin D.C., Assenov Y., Plass C., Koeffler H.P. (2018). Maftools: Efficient and comprehensive analysis of somatic variants in cancer. Genome Res..

[54] Tomczak K., Czerwinska P., Wiznerowicz M. (2015). The Cancer Genome Atlas (TCGA): An immeasurable source of knowledge. Contemp. Oncol..

[55] Goldman M.J., Craft B., Hastie M., Repečka K., McDade F., Kamath A., Banerjee A., Luo Y., Rogers D., Brooks A.N. (2020). Visualizing and interpreting cancer genomics data via the Xena platform. Nat. Biotechnol..

[56] Yu G., He Q.Y. (2016). ReactomePA: An R/Bioconductor package for reactome pathway analysis and visualization. Mol. Biosyst..

[57] Hsu C.L., Ou D.L., Bai L.Y., Chen C.W., Lin L., Huang S.F., Cheng A.L., Jeng Y.M., Hsu C. (2021). Exploring Markers of Exhausted CD8 T Cells to Predict Response to Immune Checkpoint Inhibitor Therapy for Hepatocellular Carcinoma. Liver Cancer.

[58] Hwang S., Kwon A.Y., Jeong J.Y., Kim S., Kang H., Park J., Kim J.H., Han O.J., Lim S.M., An H.J. (2020). Immune gene signatures for predicting durable clinical benefit of anti-PD-1 immunotherapy in patients with non-small cell lung cancer. Sci. Rep..

[59] Van Allen E.M., Miao D., Schilling B., Shukla S.A., Blank C., Zimmer L., Sucker A., Hillen U., Foppen M.H.G., Goldinger S.M. (2015). Genomic correlates of response to CTLA-4 blockade in metastatic melanoma. Science.

[60] Rizvi N.A., Hellmann M.D., Snyder A., Kvistborg P., Makarov V., Havel J.J., Lee W., Yuan J.D., Wong P., Ho T.S. (2015). Mutational landscape determines sensitivity to PD-1 blockade in non-small cell lung cancer. Science.

[61] McDermott D.F., Huseni M.A., Atkins M.B., Motzer R.J., Rini B.I., Escudier B., Fong L., Joseph R.W., Pal S.K., Reeves J.A. (2018). Clinical activity and molecular correlates of response to atezolizumab alone or in combination with bevacizumab versus sunitinib in renal cell carcinoma. Nat. Med..

[62] Liu D., Schilling B., Liu D., Sucker A., Livingstone E., Jerby-Arnon L., Zimmer L., Gutzmer R., Satzger I., Loquai C. (2019). Integrative molecular and clinical modeling of clinical outcomes to PD1 blockade in patients with metastatic melanoma. Nat. Med..

[63] Riaz N., Havel J.J., Makarov V., Desrichard A., Urba W.J., Sims J.S., Hodi F.S., Martin-Algarra S., Mandal R., Sharfman W.H. (2017). Tumor and Microenvironment Evolution during Immunotherapy with Nivolumab. Cell.

[64] Kim S.T., Cristescu R., Bass A.J., Kim K.M., Odegaard J.I., Kim K., Liu X.Q., Sher X., Jung H., Lee M. (2018). Comprehensive molecular characterization of clinical responses to PD-1 inhibition in metastatic gastric cancer. Nat. Med..

[65] Lee J.H., Shklovskaya E., Lim S.Y., Carlino M.S., Menzies A.M., Stewart A., Pedersen B., Irvine M., Alavi S., Yang J.Y.H. (2020). Transcriptional downregulation of MHC class I and melanoma de- differentiation in resistance to PD-1 inhibition. Nat. Commun..

[66] Nathanson T., Ahuja A., Rubinsteyn A., Aksoy B.A., Hellmann M.D., Miao D., Van Allen E., Merghoub T., Wolchok J.D., Snyder A. (2017). Somatic Mutations and Neoepitope Homology in Melanomas Treated with CTLA-4 Blockade. Cancer Immunol. Res..

[67] Snyder A., Nathanson T., Funt S.A., Ahuja A., Buros Novik J., Hellmann M.D., Chang E., Aksoy B.A., Al-Ahmadie H., Yusko E. (2017). Contribution of systemic and somatic factors to clinical response and resistance to PD-L1 blockade in urothelial cancer: An exploratory multi-omic analysis. PLoS Med..

[68] Gide T.N., Quek C., Menzies A.M., Tasker A.T., Shang P., Holst J., Madore J., Lim S.Y., Velickovic R., Wongchenko M. (2019). Distinct Immune Cell Populations Define Response to Anti-PD-1 Monotherapy and Anti-PD-1/Anti-CTLA-4 Combined Therapy. Cancer Cell.

[69] Braun D.A., Hou Y., Bakouny Z., Ficial M., Sant’ Angelo M., Forman J., Ross-Macdonald P., Berger A.C., Jegede O.A., Elagina L. (2020). Interplay of somatic alterations and immune infiltration modulates response to PD-1 blockade in advanced clear cell renal cell carcinoma. Nat. Med..

[70] Kim J.Y., Choi J.K., Jung H. (2020). Genome-wide methylation patterns predict clinical benefit of immunotherapy in lung cancer. Clin. Epigenetics.

[71] Jung H., Kim H.S., Kim J.Y., Sun J.M., Ahn J.S., Ahn M.J., Park K., Esteller M., Lee S.H., Choi J.K. (2019). DNA methylation loss promotes immune evasion of tumours with high mutation and copy number load. Nat. Commun..

[72] Miao D., Margolis C.A., Gao W., Voss M.H., Li W., Martini D.J., Norton C., Bossé D., Wankowicz S.M., Cullen D. (2018). Genomic correlates of response to immune checkpoint therapies in clear cell renal cell carcinoma. Science.

[73] Sun S., Xu L., Zhang X., Pang L., Long Z., Deng C., Zhu J., Zhou S., Wan L., Pang B. (2021). Systematic Assessment of Transcriptomic Biomarkers for Immune Checkpoint Blockade Response in Cancer Immunotherapy. Cancers.

[74] Lin A., Qi C., Wei T., Li M., Cheng Q., Liu Z., Luo P., Zhang J. (2022). CAMOIP: A web server for comprehensive analysis on multi-omics of immunotherapy in pan-cancer. Brief. Bioinform..

[75] Li Y., Burgman B., McGrail D.J., Sun M., Qi D., Shukla S.A., Wu E., Capasso A., Lin S.Y., Wu C.J. (2020). Integrated Genomic Characterization of the Human Immunome in Cancer. Cancer Res..

[76] Wang K., Patkar S., Lee J.S., Gertz E.M., Robinson W., Schischlik F., Crawford D.R., Schaffer A.A., Ruppin E. (2022). Deconvolving clinically relevant cellular immune crosstalk from bulk gene expression using CODEFACS and LIRICS stratifies melanoma patients to anti-PD-1 therapy. Cancer Discov..

[77] Chen Y., Jia K., Sun Y., Zhang C., Li Y., Zhang L., Chen Z., Zhang J., Hu Y., Yuan J. (2022). Predicting response to immunotherapy in gastric cancer via multi-dimensional analyses of the tumour immune microenvironment. Nat. Commun..

[78] Holm J.S., Funt S.A., Borch A., Munk K.K., Bjerregaard A.-M., Reading J.L., Maher C., Regazzi A., Wong P., Al-Ahmadie H. (2022). Neoantigen-specific CD8 T cell responses in the peripheral blood following PD-L1 blockade might predict therapy outcome in metastatic urothelial carcinoma. Nat. Commun..

[79] Valpione S., Galvani E., Tweedy J., Mundra P.A., Banyard A., Middlehurst P., Barry J., Mills S., Salih Z., Weightman J. (2020). Immune-awakening revealed by peripheral T cell dynamics after one cycle of immunotherapy. Nat. Cancer.

[80] Wu T.D., Madireddi S., de Almeida P.E., Banchereau R., Chen Y.J., Chitre A.S., Chiang E.Y., Iftikhar H., O’Gorman W.E., Au-Yeung A. (2020). Peripheral T cell expansion predicts tumour infiltration and clinical response. Nature.

