# Supplementary material for: A Comprehensive Benchmark of Transcriptomic Biomarkers for Immune Checkpoint Blockades
Source: Cancers (Basel). 2023 Aug 14;15(16):4094. doi: 10.3390/cancers15164094 (PMC10452274; doi:10.3390/cancers15164094)
Supplement: Supplementary file 1 [file cancers-15-04094-s001.zip › Supplementary Figure S2.pdf]

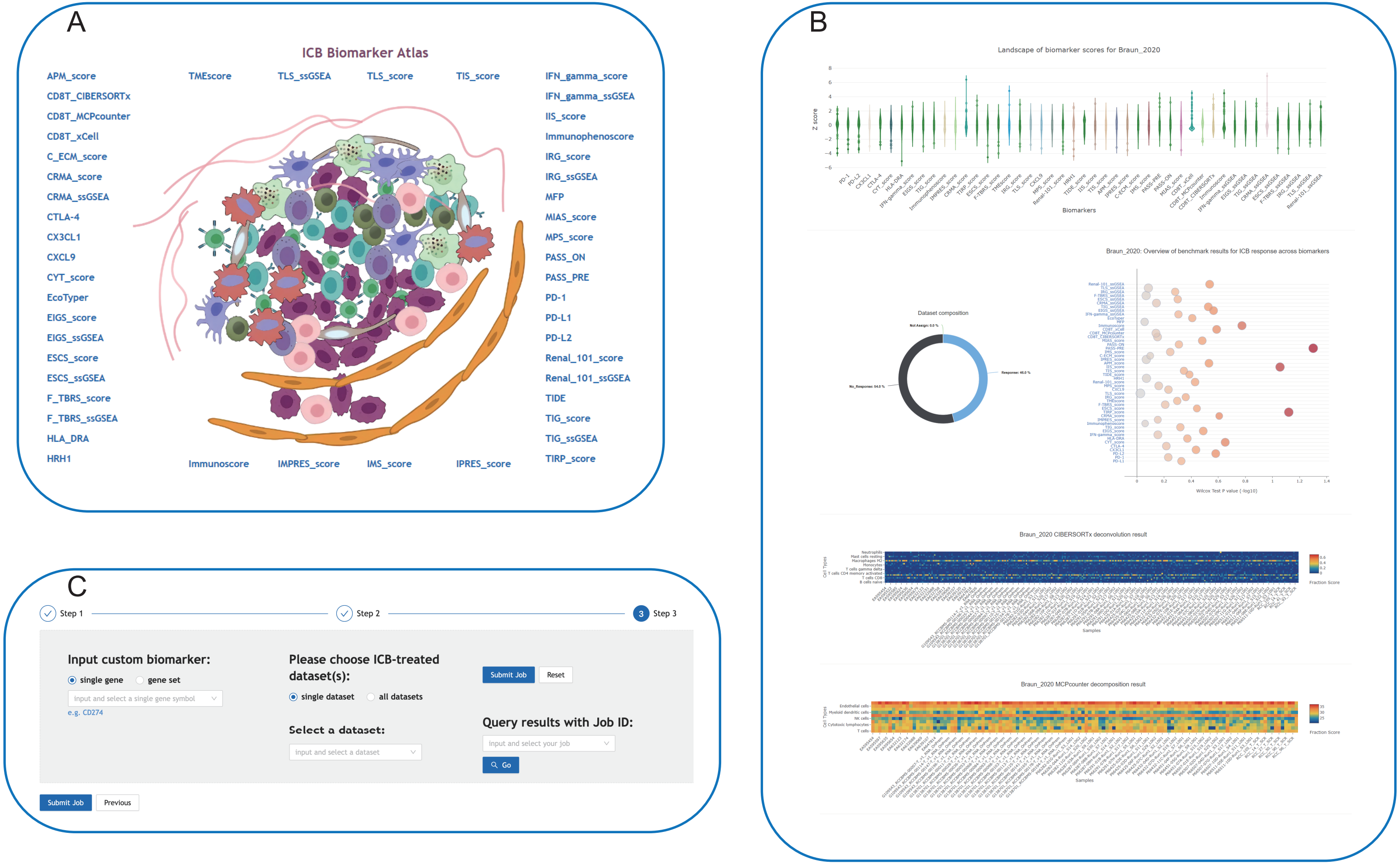
A
ICB Biomarker Atlas
APM_score
CD8T_CIBERSORTx
CD8T_MCPcounter
CD8T_xCell
C_ECM_score
CRMA_score
CRMA_ssGSEA
CTLA-4
CX3CL1
CXCL9
CYT_score
EcoTyper
EIGS_score
EIGS_ssGSEA
ESCS_score
ESCS_ssGSEA
F_TBRS_score
F_TBRS_ssGSEA
HLA_DRA
HRH1
TMEscore
TLS_ssGSEA
TLS_score
TIS_score
Immunoscore
IMPRES_score
IMS_score
IPRES_score
IFN_gamma_score
IFN_gamma_ssGSEA
IIS_score
Immunophenoscore
IRG_score
IRG_ssGSEA
MFP
MIAS_score
MPS_score
PASS_ON
PASS_PRE
PD-1
PD-L1
PD-L2
Renal_101_score
Renal_101_ssGSEA
TIDE
TIG_score
TIG_ssGSEA
TIRP_score
B
Landscape of biomarker scores for Braun_2020
Z score
Biomarkers
Braun_2020: Overview of benchmark results for ICB response across biomarkers
Dataset composition
Not Assign: 0.0 %
Response: 46.0 %
No_Response: 54.0 %
Wilcox Test P value (-log10)
Braun_2020 CIBERSORTx deconvolution result
Cell Types
Samples
Fraction Score
Braun_2020 MCPcounter decomposition result
Cell Types
Samples
Fraction Score
C
Step 1
Step 2
3 Step 3
Input custom biomarker:
single gene
gene set
input and select a single gene symbol
e.g. CD274
Please choose ICB-treated dataset(s):
single dataset
all datasets
Select a dataset:
input and select a dataset
Submit Job
Reset
Query results with Job ID:
input and select your job
Go
Submit Job
Previous
