# Supplementary figures and images for: A Comprehensive Benchmark of Transcriptomic Biomarkers for Immune Checkpoint Blockades
Source: Cancers (Basel). 2023 Aug 14;15(16):4094. doi: 10.3390/cancers15164094 (PMC10452274; doi:10.3390/cancers15164094)

# Biomarker

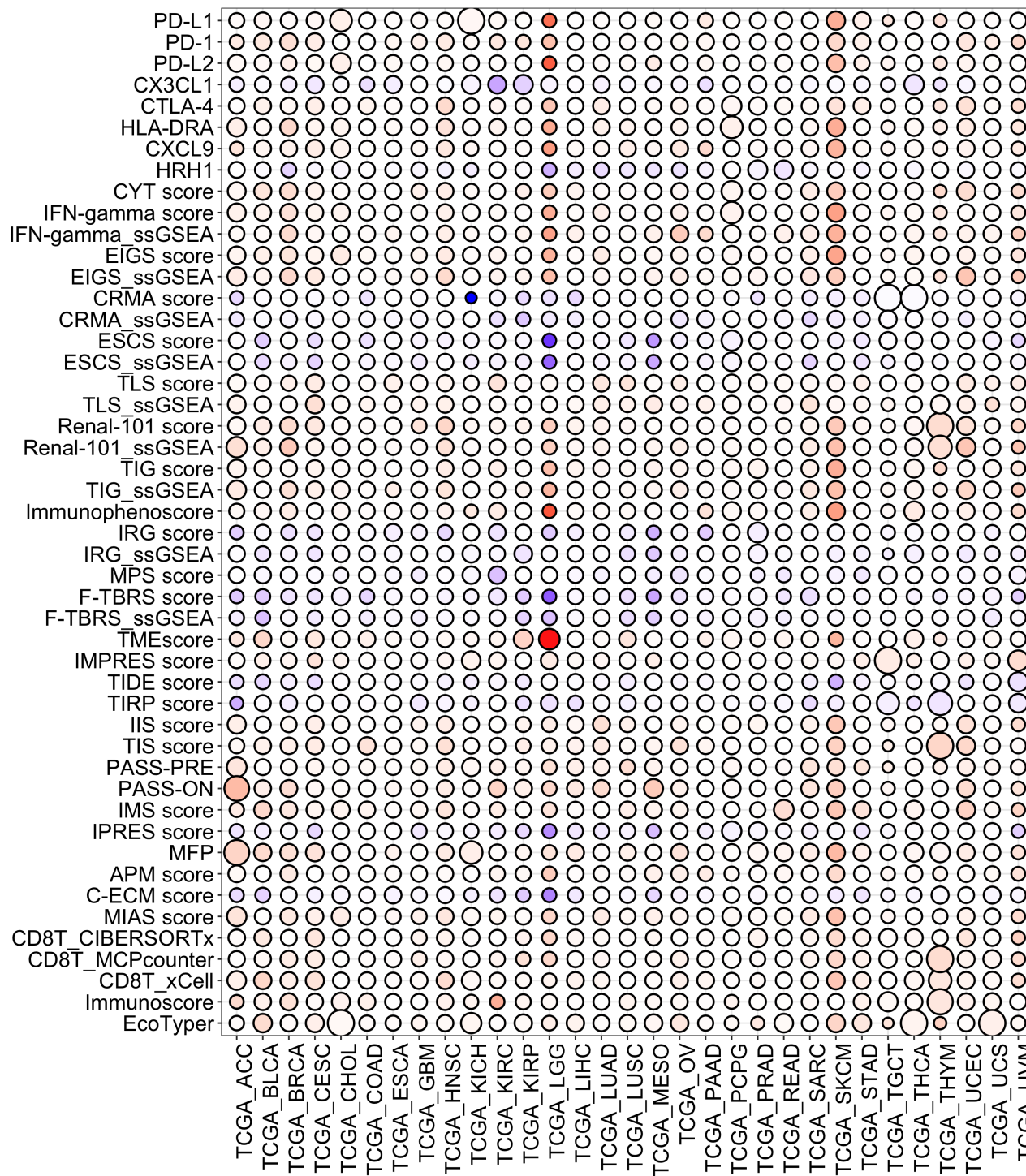

HR

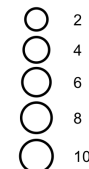

$-\log_{10}(p)$

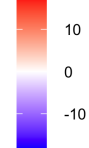

Statistics

Dataset

Supplement: Supplementary file 1 [file cancers-15-04094-s001.zip › Supplementary Figure S1.pdf]
